# Elevated Serum Soluble CD163 Indicates Macrophage Activation: A Potential Biomarker for Inflammation in Complex Regional Pain Syndrome?

**DOI:** 10.1111/papr.70189

**Published:** 2026-07-17

**Authors:** Nadia Kriek, Krishna Bharwani, Maaike Dirckx, Dirk Stronks, Nicole Nagtzaam, Wim A. Dik, Frank Huygen

**Affiliations:** ^1^ Center for Pain Medicine, Department of Anesthesiology Erasmus MC University Medical Center Rotterdam the Netherlands; ^2^ Laboratory Medical Immunology, Department of Immunology Erasmus MC University Medical Center Rotterdam the Netherlands

## Abstract

**Background:**

Several studies point towards the involvement of the immune system (especially the monocyte–macrophage system) in the pathophysiology of Complex Regional Pain Syndrome (CRPS), as shown by increased levels of the cytokines tumor necrosis factor (TNF)‐α and interleukin (IL)‐6. These cytokines are primarily released from pro‐inflammatory M1 macrophages, but are difficult to measure in a clinical setting. Investigating the activity of tissue‐resident macrophages by measuring soluble CD163 (sCD163) in serum is much more clinically applicable. sCD163 could potentially be a biomarker for determining inflammation in CRPS and would have direct consequences for the choice of therapy. The aim of this study is to investigate the tissue‐resident macrophage activation in CRPS with sCD163 and explore the relationship between soluble IL‐2R, which is a marker for T‐cell activation, and sCD163.

**Methods:**

The sCD163 levels were determined in this retrospective cohort study (MEC‐2020‐0716) in serum of CRPS patients (*n* = 22) and healthy controls (*n* = 27). ELISA kits were used to measure the levels of these markers. Data on demographics, pain scores (11‐point Numeric Rating Scale: NRS), sIL‐2R levels, signs and symptoms, and CRPS disease severity were also recorded.

**Results:**

The median serum sCD163 level was significantly higher in CRPS patients (CRPS 960.5 pg/mL [Q3–Q1: 1211.5–623.9] than in healthy controls 677.0 pg/mL [Q3–Q1: 828.0–469.5], *p* = 0.008). There was a statistically significant positive correlation between sCD163 and sIL‐2R in the CRPS group (*r*_*s*_ = 0.577; *p* = 0.005), but not in the healthy control group (*r*_*s*_ = 0.359; *p* = 0.066).

**Conclusion:**

Our findings indicate that pro‐inflammatory activation of tissue‐resident macrophages and thus the monocyte–macrophage system is part of CRPS pathogenesis. The monocyte–macrophage system is an interesting new target for future research into the pathogenesis of CRPS, but also has potential for diagnosis, particularly for assessing the involvement of the immune system and choice of therapy in the individual CRPS patient. Measurement of the sCD163 could be a potential biomarker for inflammation in CRPS.

**Trial Registration:** This retrospective cohort study was approved by the Medical Ethics Committee of the Erasmus MC University Medical Center (MEC‐2020‐0716).

## 1 Background

Complex Regional Pain Syndrome (CRPS) is primarily a limb‐confined syndrome that is mostly preceded by trauma to the affected limb. It is characterized by continuous pain in the affected limb which is accompanied by two or more sensory, motor, vasomotor, sudomotor, and trophic disturbances [1]. CRPS is diagnosed using the new clinical diagnostic criteria from the International Association for the Study of Pain (IASP) [1]. There are still no objective biomarkers or tests to diagnose this disease. The treatment of CRPS is conducted in a mechanism‐based manner: it targets the active pathophysiological mechanisms in each CRPS case [2].

The pathophysiology of CRPS is still incompletely understood. It is generally accepted that multiple mechanisms play a role in the onset and maintenance of CRPS [3]. An important pathophysiological mechanism in CRPS is dysregulation of both the innate and adaptive branch of the immune system [4]. Involvement of the adaptive immune system in CRPS is suggested by a number of observations including a higher prevalence of serum autoantibodies against autonomic nervous system autoantigen and anti‐nuclear antibodies [5, 6, 7, 8], significantly lower peripheral blood numbers of cytotoxic CD8^+^ T cells and Th17 cells, increased peripheral blood proportions of CD39^+^Tregs and elevated serum levels of the T‐lymphocyte activation marker soluble interleukin‐2 receptor (sIL‐2R) [9, 10, 11]. The measurement in serum of the biomarker sIL‐2R is already applied in other auto‐inflammatory diseases like rheumatoid arthritis and sarcoidosis. In the later it is used as a disease marker and for monitoring the efficacy of immunemodulating drugs like anti‐TNF. In CRPS, the biomarker sIL‐2R can be used to measure the involvement of inflammation as underlying mechanism and can guide in therapy choice, making therapy much more mechanism based and personalized. Involvement of the innate immune system in CRPS is mostly supported by elevated levels of the innate cytokines tumor necrosis factor (TNF)‐α and interleukin (IL)‐6 in fluid from artificially made skin blisters of the CRPS affected limbs, but also by increased circulating CD14^+^CD16^+^ pro‐inflammatory monocytes [12, 13, 14]. These latter data point towards monocytes, and their descendent tissue‐resident macrophages, as important cellular components of the (immuno‐)pathophysiology of CRPS. This is further supported by studies that indicate that the monocyte–macrophage compartment may be a valuable target for therapy in CRPS. Studies have shown that Thalidomide, a potent immunomodulatory and anti‐inflammatory drug with inhibitory effects on TNF‐α production (especially from monocytes/macrophages), seems to be effective in a subgroup of CRPS patients [15, 16, 17, 18]. Therefore, measuring activity of the monocyte–macrophage system could be of value in the diagnostic work‐up, as well as monitoring of disease activity and therapy efficacy in CRPS.

Although alterations in peripheral blood monocyte subset distribution can be indicative of inflammatory activation, it does not necessarily reflect a pathological activation of macrophages within the tissues [19]. Tissue‐resident macrophages are important immune cells in maintaining tissue homeostasis and can react rapidly to disturbances in homeostasis, as seen during physiological stress or infectious events. At sites of tissue injury, macrophages orchestrate the local inflammatory response and are responsible for scavenging cellular debris and mediating tissue repair [19, 20]. Not much is known on the contribution of tissue resident macrophages to CRPS. However, a recent study used the chronic post ischemia pain model (CPIP) of CRPS in mice and demonstrated a role for macrophages in sustaining chronic neuroinflammation and allodynia [21]. Although necessary caution must be exercised when extrapolating findings from animal models to humans, these findings, together with previously mentioned observations, warrant further investigation into the role of macrophages in the pathophysiology of CRPS. Assessing the activation of tissue‐resident macrophages could lead to a better understanding on the role of the monocyte–macrophage compartment in the (local) inflammatory processes that takes place in CRPS.

Examination of tissue‐resident macrophages in CRPS is hampered by the fact that the process of obtaining tissue specimens is perceived as uncomfortable by patients, labor intensive for physicians and laboratories and this process may risk reactivation/aggravation of the syndrome. Furthermore, there are limited numbers of histopathological CRPS studies and, to our knowledge, none of them have explored the macrophages [22, 23, 24]. Yet, the supposed contribution of macrophages to CRPS pathophysiology warrants further investigation on this topic and preferably by using minimally invasive measures. Circulating sCD163 is a systemic marker of pro‐inflammatory activation of tissue‐resident macrophages, which is easily measurable in serum. Soluble CD163 is the circulating, extracellular portion of the membrane receptor CD163 [25], which is the high affinity scavenger receptor for haptoglobin‐hemoglobin complexes. This receptor is solely expressed on monocytes and macrophages. Upon activation by pro‐inflammatory stimuli, CD163 is enzymatically cleaved from the macrophage surface by ADAM17/TACE (tumor necrosis factor α‐converting enzyme), the enzyme that also releases TNF‐α into the circulation [25, 26, 27]. In healthy individuals, circulating levels of sCD163 are low, but during inflammation and macrophage activation, levels of sCD163 increase rapidly and substantially [25]. Soluble CD163 is now considered a useful biomarker of macrophage activation in various inflammatory and infectious conditions and may correlate with disease activity in diseases such as rheumatoid arthritis and sarcoidosis [25, 28, 29, 30, 31]. Potentially it could be, besides the earlier identified biomarker sIL2‐R, a second biomarker in CRPS to identify underlying inflammation, which has direct consequences for choice of therapy.

In this study, we investigated whether there are signs of tissue‐resident macrophage activation in CRPS by measuring the serum sCD163 levels in venous blood of CRPS patients and in healthy controls. Furthermore, we explored the relation between sCD163 and sIL‐2R in CRPS.

## 2 Methods

### 2.1 Ethical Approval and Patient Consent

This retrospective cohort study, with reference number (MEC‐2020‐0716), was reviewed by ethics committee/Institutional Review Board (IRB) of the Medical Ethics Committee Erasmus MC of Rotterdam, The Netherlands on the 30th of September 2020. As a result of this review, the Committee has declared that the rules laid down in the Medical Research Involving Human Subjects Act (also known by its Dutch abbreviation WMO), do not apply to this research proposal and therefore an exemption was granted from requiring ethics approval.

The study itself was performed in accordance with the Declaration of Helsinki. All participants from which the materials were obtained had completed written informed consent prior to enrolling in this study. A Clinical trial number is not applicable.

### 2.2 Study Population and Data Collection

The study population consists of patients with CRPS in one limb who were diagnosed at the Center for Pain Medicine by using the new IASP clinical diagnostic criteria for CRPS [1], along with a group of healthy controls.

Venous blood samples of CRPS patients and healthy controls were collected during previous studies in our institution that were conducted at the Center for Pain Medicine (MEC‐2017‐495) [32] and the Laboratory Medical Immunology (MEC‐2016‐202) [28]. All participants included for analysis in this current study signed informed consent forms declaring that their data and blood samples could be stored and used for future research.

For the CRPS group, data on demographics, pain scores (11‐point Numeric Rating Scale: NRS), sIL‐2R levels, signs and symptoms, and CRPS disease severity as measured using the CRPS severity score‐Database Form [33] were available from our previous study [32]. sCD163 and sIL‐2R levels of healthy controls were also available from a previous study and used for the analysis in this study (MEC‐2016‐202) [28]. Demographic data of the healthy controls were unavailable for analysis in this study.

### 2.3 Serum sCD163 and sIL‐2R Analysis

Serum sCD163 was measured in CRPS patients with ELISA (Trillium Diagnostics/IQ Products BV, Groningen, the Netherlands) according to the manufacturer's instructions, without modification. Results are expressed in picograms/mL (pg/mL). Reference values for serum sCD163 levels in healthy controls were previously obtained using the same assay [28].

Serum sIL‐2R levels (measured with ELISA; Diaclone, Besancon, France) of the CRPS patients and healthy controls were available from our previous studies (MEC‐2017‐495 [32]; MEC‐2016‐202 [28]) and were used for analysis in this study. Serum sCD163 and sIL‐2R measurements were conducted according to the manufacturer's instructions, within the diagnostic facility of the Laboratory Medical Immunology, Erasmus MC Rotterdam, and under strict quality regulations (ISO15189).

### 2.4 Statistical Analysis

Descriptive statistics were used to determine the frequencies of categorical variables and to describe measures of central tendency and variability of continuous variables. A Shapiro–Wilk test was used to analyze whether or not continuous variables were normally distributed.

The primary outcome parameter of this study was the sCD163 level in CRPS patients compared to the healthy control group. Secondary outcomes were (1) the association between sCD163 levels and sIL‐2R levels between and within both groups and (2) the association between sCD163 level and CRPS severity score in the CRPS group.

Dependent on the shape of their distribution, continuous variables were compared across the CRPS group and healthy control group by using either an independent samples *T*‐test or an independent samples Mann–Whitney *U*‐test. Categorical variables were compared using a Fisher's exact test.

The association between sCD163 level and sIL‐2R level and sCD163 level and CRPS severity score was explored using either a Pearson correlation or Spearman's rank correlation depending on the shape of the distribution of these variables. In addition, in CRPS patients, the association between sCD163 and age, duration of disease and pain scores at the time of visit was also explored using either a Pearson correlation or Spearman's rank correlation also depending on the shape of the distribution of these variables. The association between sCD163 and gender was explored using a point‐biserial correlation.

The central tendency and dispersion of the variables with a normal distribution are expressed in their means and standard deviations (SD). Concerning the variables with a skewed distribution these parameters are expressed in medians and interquartile ranges (Q3–Q1). Categorical variables are expressed in frequencies. Analyses were conducted using IBM SPSS Statistics 25. The alpha level for statistical significance was set at 0.05.

## 3 Results

Serum samples obtained in previous studies were available from 22 CRPS patients and 27 healthy controls. One blood sample from the original 23 patients in the CRPS group was missing. The characteristics of the CRPS group are presented in Table 1.

**TABLE 1:** Characteristics of CRPS group.

	CRPS group (*n* = 22)
Age in years (median [Q3–Q1])	42.5 (55.5–29.5)
Duration of disease in months (median [Q3–Q1])	26.0 (81.8–14.8)
Gender (*n* [%])	
Male	4 (18.2)
Female	18 (81.8)
NRS at time of visit (mean [SD])	7.1 (1.25)
Precipitating injury (*n* [%])	
Trauma	10 (45.5)
Operation	9 (40.9)
Other	2 (9.1)
Unknown	1 (4.5)
Affected limb (*n* [%])	
Right upper limb	4 (18.2)
Right lower limb	4 (18.2)
Left upper limb	4 (18.2)
Left lower limb	10 (45.5)
Temperature of affected limb during physical examination (*n* [%])	
Warm	0
Cold	7 (31.8%)
No difference	15 (68.2%)
CRPS severity score (mean [SD])	11.2 (1.97)

**FIGURE 1:**
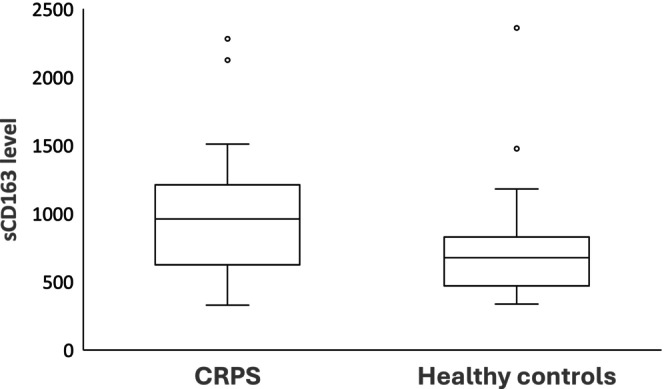
Boxplot of serum sCD163 level in the CRPS and the healthy control group. The median sCD163 level in the CRPS group 960.5 pg/mL (Q3–Q1: 1211.5–623.9) versus the healthy control group 677.0 pg/mL (Q3–Q1: 828.0–469.5), *p* = 0.008.

The median sCD163 level of the CRPS group was significantly higher than that of the healthy control group: CRPS 960.5 pg/mL (Q3–Q1: 1211.5–623.9) versus healthy controls 677.0 pg/mL (Q3–Q1: 828.0–469.5), *p* = 0.008 (Figure 1). Median sIL‐2R level was 2889.5 pg/mL (Q3–Q1: 3960.8–1592.8) in the CRPS group versus 2115.0 pg/mL (Q3–Q1: 2842.0–1757.0) in the healthy control group [28, 32].

**FIGURE 2:**
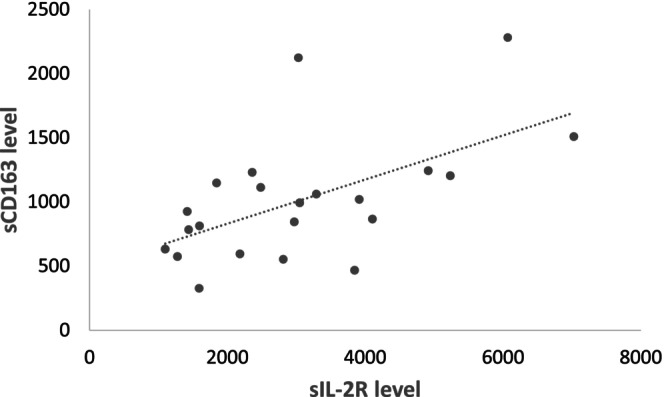
Scatterplot of the serum sIL‐2R and sCD163 level in the CRPS group (*r*_*s*_ = 0.577; *p* = 0.005). Association between sCD163 (pg/mL) and sIL‐2R (pg/mL) revealed a statistically significant positive correlation in the CRPS group (*r*_*s*_ = 0.577; *p* = 0.005), but not in the healthy control group (*r*_*s*_ = 0.359; *p* = 0.066).

There was a statistically significant positive correlation between sCD163 and sIL‐2R in the whole study population that is, the CRPS group and healthy controls: *r*_*s*_ = 0.512, *p* < 0.001. Further analysis of this association between sCD163 and sIL‐2R revealed a statistically significant positive correlation in only the CRPS group (*r*_*s*_ = 0.577; *p* = 0.005), as shown in Figure 2, but not in the healthy control group (*r*_*s*_ = 0.359; *p* = 0.066).

In the CRPS group, no association was found between sCD163 and the CRPS severity score: *r*_*s*_ = −0.324, *p* = 0.14. No association was found in the CRPS group between sCD163 and age (*r*_*s*_ = 0.040, *p* = 0.86), gender (*r*_*s*_ = −0.037, *p* = 0.87), durations of signs and symptoms (*r*_*s*_ = −0.390, *p* = 0.07) and pain score at the time of visit (*r*_*s*_ = 0.060, *p* = 0.79).

## 4 Discussion

In this study, we explored whether there are signs of activation of tissue‐resident macrophages by means of measuring serum sCD163 levels in CRPS patients and comparing them to healthy controls. The results from this study showed that serum sCD163 levels were significantly higher in CRPS patients than in healthy controls. This indicates enhanced activity of tissue‐resident macrophages and further supports involvement of the monocyte–macrophage system in the pathophysiology of CRPS. In addition, serum sCD163 and sIL‐2R levels displayed a significant positive correlation in the CRPS group. Taken together, our findings support concurrent activation of the innate and adaptive branches of the immune system in CRPS.

To our knowledge, our study is the first to demonstrate that CRPS is associated with elevated serum sCD163, indicating that elevated macrophage activity is associated with CRPS. Although the trigger(s) that establish macrophage activation in CRPS remain unknown, our finding of enhanced macrophage activity in CRPS are corroborated by several other observations. First, levels of TNF‐α and IL‐6, which are cytokines primarily released from local pro‐inflammatory M1 macrophages [34, 35], have been found to be elevated in blister fluid of the affected limb in CRPS patients [12]. Notably, sCD163 is cleaved from the macrophage membrane upon activation by pro‐inflammatory stimuli by the enzyme ADAM17/TACE (tumor necrosis factor α‐converting enzyme), the same enzyme that releases TNF‐α into the circulation [25, 26]. Second, an increase in circulating levels of the pro‐inflammatory CD14^+^CD16^+^ monocytes in CRPS patients has been found [14]. CD14^+^CD16^+^ monocytes produce high levels of pro‐inflammatory cytokines such as TNF‐α and display the highest CD163 surface membrane expression of the main circulating monocyte subsets, while pro‐inflammatory activation can facilitate active shedding of CD163 from human monocytes [36, 37, 38, 39]. Third, Thalidomide, a drug that inhibits TNF‐α production by monocytes, exerts beneficial clinical effects in a subgroup of CRPS patients [15, 16, 18]. Thus, our results strengthen the notion that elevated macrophage and monocyte activity is involved in the pathophysiology of CRPS.

Although we did not find a statistically significant correlation between sCD163 and CRPS disease severity in our CRPS group, we observed that the correlation that was found was a weak downhill (i.e., negative) correlation. Despite not being statistically significant, this finding is in line with findings in our previous study in which a significant negative correlation was shown between serum sIL‐2R levels and CRPS disease severity [32]. Although not part of our primary results, we performed a post hoc analysis of the correlation of sIL‐2R and CRPS disease severity and also found a moderate negative correlation, although statistical significance was not achieved possibly due to this study being underpowered for this result: *r*_*s*_ = −0.422, *p* = 0.051 (a posteriori power [1‐β] = 0.47). We previously proposed, based on this negative correlation between sIL‐2R and CRPS disease severity, that sIL‐2R may be a marker for inflammatory disease activity (the intensity of the inflammatory process) and not disease severity (the damage caused by this inflammatory process) and consequently, that the level of elevation may be related to the phase of disease (i.e., acute vs. chronic) [32]. This hypothesis may also be applicable to our current findings on serum sCD163 that may reflect inflammatory disease activity and not necessarily disease severity in CRPS. This hypothesis needs further validation studies with measurements of serum sCD163 and sIL‐2R in early acute stage of CRPS along with longitudinal follow‐up into the chronic CRPS stage.

We further found a statistically significant moderate positive correlation between sCD163 and sIL‐2R in our CRPS group. This finding indicates activation of both the monocyte–macrophage compartment and T‐cell compartment in CRPS. As far as we are aware, our study is the first study that determined both serum sCD163 and sIL‐2R levels in a cohort of CRPS patients and the first study to demonstrate that activation of both monocytes‐macrophages and T‐cells co‐occur in CRPS patients. Our findings therefore strongly support previous notions that both (auto)inflammatory and autoimmune features contribute to CRPS disease pathogenesis [4].

Taken together, the current study shows that CRPS is associated with activation of the monocyte–macrophage system, as indicated by the elevated serum level of sCD163. These activated macrophages and monocytes are considered to contribute to inflammatory disease pathology in CRPS and may therefore represent a new research area on pathogenesis, diagnosis and management of CRPS. In addition, the findings from this study warrant further exploration of sCD163 as a potential new biomarker for diagnosis and therapy in CRPS. As this marker can easily be measured in blood, it is less invasive than gathering tissue specimens to analyze macrophage or monocyte activation. Our observation of concurrent activation of the innate and adaptive branches of the immune system may be an indication that optimal control of CRPS inflammation requires clinical therapies that target both innate and adaptive immune activation. However, future research is still needed to explore the use of serum sCD163 and sIL‐2R for therapeutic stratification in CRPS. Serum sIL‐2R is not a suitable biomarker to diagnose CRPS from other pain conditions, at least in a tertiary care setting [32]. For serum sCD163 this is yet unknown and therefore studies need to be conducted to explore the value of serum sCD163 in diagnosing CRPS.

Our study is not without limitations and the various (methodological) weaknesses are summarized below.

(1) This is a retrospective cohort study with a single serum sCD163 measurement in CRPS patients with a relatively long disease duration that is, chronic (cold) type CRPS. Thus, conclusions cannot be drawn on changes in serum sCD163 level during the disease course of the syndrome since there are no samples of the same CRPS patients during the acute phase. Though, patients in the acute phase of CRPS that is, with a short syndrome duration, often have an inflammatory phenotype [3]. It is likely that serum sCD163 levels of CRPS patients in the acute phase may differ from the levels of our group of chronic CRPS patients.

(2) sCD163 is a non‐specific marker whose levels are elevated in various inflammatory conditions and infectious diseases. Only sCD163 for monocyte–macrophage activation and sIL‐2R for T‐cell activation were measured. Therefore, this study does not reflect the full spectrum of activation of the various different monocyte–macrophage and T‐cell subsets in CRPS.

(3) Due to the retrospective nature of this study, we were not able to correlate our findings of the systemic involvement of the innate and adaptive immune system, as measured by sCD163 and sIL‐2R respectively, with the regional inflammatory processes as reflected by blister fluids [12, 13, 40, 41, 42].

(4) No demographic data were available for the healthy control group and thus patients and controls could not be matched based on age, gender or other (metabolic) factors.

(5) In this study we compare material from patients with CRPS to that from healthy volunteers. An important step still needed in the validation process is to compare material from CRPS patients with that from patients who have another distal disorder of the extremities, such as carpal tunnel syndrome. This is a step to go in a new prospective study, although it remains uncertain whether a comparable patient group for CRPS exists.

(6) For a relatively rare disease such as CRPS, a sample size of *n* = 22 is still quite small, which limits its statistical power and increases the risk of both type I and type II errors.

## 5 Conclusion

Soluble CD163 is increased in CRPS patients compared to healthy controls. This finding suggests activation of the monocyte–macrophage system in CRPS. Furthermore, our observation that activation of the innate immune system is accompanied by activation of the adaptive immune system warrants further research into the role and interactions between these two branches of the immune system and into the pathophysiology of CRPS.

The monocyte‐macrophages system and the sCD163 marker may therefore represent an interesting new target for diagnosing CRPS, distinguishing the inflammatory CRPS phenotype from other CRPS phenotypes, guiding the selection of (anti‐inflammatory) medication and monitoring therapy efficacy over time.

All of these aspects should be further explored in a larger prospective study in which various biomarkers, such as sCD163 and sIL2‐r are measured in CRPS patients and compared with both healthy controls and other inflammatory/distal extremity disorders at different time‐points, so that both the acute and chronic phases are taken into account, and guidance can be provided for therapeutic management.

## Author Contributions

Nadia Kriek and Krishna Bharwani are both co‐first authors of this manuscript. All authors contributed equally to the study design, execution, analyses and manuscript writing. The final manuscript was read, corrected and approved by all authors before submission.

## Funding

The authors have nothing to report.

## Ethics Statement

This retrospective cohort study, with reference number (MEC‐2020‐0716), was reviewed by the Rotterdam, The Netherlands on the 30th of September 2020. As a result of this review, the Committee has declared that the rules laid down in the Medical Research Involving Human Subjects Act (also known by its Dutch abbreviation WMO), do not apply to this research proposal and therefore an exemption was granted from requiring ethics approval.

## Consent

The study itself was performed in accordance with the Declaration of Helsinki. All participants from which the materials were obtained had completed written informed consent prior to enrolling in this study.

## Conflicts of Interest

F. Huygen is on the advisory board of Abbott, Saluda, Salvia, Pfizer and Boston Scientific. All other authors declare no conflicts of interest.

## Data Availability

The data that support the findings of this study are available on request from the corresponding author. The data are not publicly available due to privacy or ethical restrictions.
